# Morphologic change in deep venous thrombosis in the lower extremity after therapeutic anticoagulation

**DOI:** 10.1186/s12959-021-00352-0

**Published:** 2021-12-14

**Authors:** Tanapong Panpikoon, Wisanu Phattharaprueksa, Tharintorn Treesit, Chinnarat Bua-ngam, Kaewpitcha Pichitpichatkul, Apichaya Sriprachyakul

**Affiliations:** Department of Diagnostic and Therapeutic Radiology, Faculty of Medicine Ramathibodi Hospital, Mahidol University, 270 Rama VI Rd. Phyathai, Ratchathewi, 10400 Bangkok, Thailand

**Keywords:** Deep vein thrombosis, Venous duplex ultrasound, Thrombus resolution, Anticoagulant treatment

## Abstract

**Background:**

To evaluate the anticoagulant treatment response in venous thrombi with different morphologies (size, shape, and echogenicity) by measuring the change in thrombus thickness.

**Materials and methods:**

This was a retrospective cohort study of 97 lower extremity DVT patients diagnosed by venous ultrasound between March 2014 and February 2018. The demographics, clinical risk factors, anticoagulant treatment, and ultrasound findings at the first diagnosis and 2–6 months after treatment were evaluated.

**Results:**

The anticoagulant treatment with LMWH followed by VKAs showed a significant decrease in the mean maximum difference in lower extremity DVT thrombus thickness compared with VKAs alone (*P*-value < 0.001). After adjustment by treatment, the thrombi found in dilated veins showed a significant decrease in the thickness of such thrombi compared with those found in small veins: 4 mm vs. 0 mm (Coef. = 3, 95% CI: 1.9, 4.1 and *P*-value < 0.001). Anechoic and hypoechoic thrombi showed a significant decrease in the thickness compared with hyperechoic thrombi: 5 mm vs. 0 mm (Coef. = 4, 95% CI: 3.25, 4.74 and *P*-value < 0.001) and 3 mm vs. 0 mm (Coef. = 2, 95% CI: 1.34, 42.66 and *P*-value < 0.001), respectively. Concentric thrombi showed a significant decrease in thickness compared with eccentric thrombi: 4 mm vs. 0 mm (Coef. = 2, 95% CI: 1.45, 2.55 and *P*-value < 0.001).

**Conclusion:**

The anticoagulant treatment with LMWH followed by VKAs shows a significant decrease in lower extremity DVT thrombus thickness compared with VKAs alone. After adjustment by treatment, the morphologic finding of acute thrombi shows a significantly decreased thickness compared with the morphologic finding of chronic thrombi.

## Introduction

Deep venous thrombosis (DVT), which commonly occurs in the lower extremities and is associated with pulmonary embolism (PE) and referred to together as venous thromboembolism (VTE), is a significant global health burden [1]. Accurate VTE diagnosis is essential due to the morbidity and mortality associated with missed diagnoses and the potential side effects, patient inconvenience, and resource implications of anticoagulant treatment [2].

The diagnosis of VTE based on clinical manifestations alone is unreliable [3]. The D-dimer test for exclusion of DVT, followed by proximal lower extremity ultrasound, is recommended in a population with low pretest probability (PTP) of lower extremity DVT. Initial proximal lower extremity ultrasound followed by whole leg ultrasound is recommended in a population with intermediate and high PTPs of lower extremity DVT [2].

The diagnostic criteria of DVT are lack of complete cross-sectional vein compressibility or direct visualization of an intraluminal thrombus in B-mode, combined or not combined with colour-Doppler ultrasound. Initial (5–21 days following diagnosis) and long-term (3–6 months following diagnosis) treatment are mandatory for all patients with positive ultrasound for DVT. Extended treatment (beyond the first 3–6 months) is based on the risk/benefit balance of continued anticoagulation, patient compliance and preference [4]. Follow-up venous ultrasound at 3–6 months after coagulation treatment has a role in evaluating recurrent DVT. A 2- or 4-mm increase in venous diameter between two measurements of the common femoral and popliteal veins is the most validated criterion for the diagnosis of recurrent DVT [5].

In addition to thrombus visualization, ultrasound also provides morphological information on the thrombus related to the progression of the thrombus-forming process, namely, fibrin deposition in the first few days, thrombolysis within a week, and fibroblast invasion and capillary development within several weeks [6]. The varied staging of thrombi results in different responses to anticoagulant treatment. Morphological information, such as the size, shape and echogenicity of the thrombus, may be another concerning factor for initial and extended anticoagulation treatment unless the patient’s factors. However, there is no study to support this hypothesis.

The purpose of this study was to evaluate the response of anticoagulation treatment in venous thrombi with different morphologies (size, shape and echogenicity) by measuring the thrombus thickness change between the maximum thickness at the first diagnosis and that at 3–6 months after long-term treatment.

## Materials and methods

A retrospective cohort study was conducted on lower extremity DVT patients diagnosed by venous ultrasound between March 2014 and February 2018. The demographics, clinical risk factors, anticoagulation treatment, and ultrasound findings at the first diagnosis and 2–6 months after treatment were evaluated. The Institutional Review Board approved the study, and informed consent was waived.

According to the standard protocol, four consultant radiologists with a minimum of 5 years of postqualification experience performed whole leg ultrasound (proximal and distal lower extremity veins). A similar protocol was used in the first diagnosis and follow-up study after anticoagulant treatment.

The protocol of lower extremity venous ultrasound begins with cross-sectional greyscale compression of the common femoral vein (CFV), femoral vein (FV) and popliteal vein (Pop V) at 2-cm intervals. Dual images before and during compression were recorded at CFV, proximal FV, mid-FV, distal FV and the popliteal vein.

The criteria from DVT diagnosis are as follows:

1. Lack of complete compressibility
2. Visualization of an intraluminal thrombus

Suppose the intraluminal thrombus or incomplete compressibility is presented. The anterior-posterior thickness of the thrombus or the incomplete compressed vein will be measured in millimetres (mm). A colour flow image is an option for confirmation of suspicious intraluminal thrombus and complete haemodynamic information. An equivalent technical examination was performed on the calf veins. No video recording was made. A Toshiba Aplio 500 ultrasound scanner equipped (Canon Medical Systems Corporation, Japan) with a 14-MHz linear-array transducer (PLT-705BT) was used in the study.

The different thicknesses of venous thrombi between the first diagnosis and 3–6 months after the long-term treatment were remeasured by three radiologists. The mean value of the three measurements was used. The thrombus, which showed the maximum difference in thickness from one of five locations (CFV, prox-FV, mid-FV, dis-FV and the popliteal vein) and one side per patient, was selected for the statistical analysis.

The consensus from three experienced radiologists was used for the morphological classification according to the following criteria:

1. Size (Fig. 1)

- Increased size; the affected vein is more than two times larger than the adjacent artery.
- Normal size: the affected vein is equal to or not more than two times larger than the adjacent artery.
- Decreased size; the affected vein is smaller than the adjacent artery.

2. Echogenicity (Fig. 2)

- Anechoic; the thrombus appears black on the screen.
- Hypoechoic; the thrombus appears grey on the screen.
- Hyperechoic; the thrombus appears white on the screen

3. Shape (Fig. 3)

- Concentric: the presence of an acute angle between the thrombus and venous wall or total occlusion
- Eccentric: the presence of an obtuse angle between the thrombus and venous wall.

**Fig. 1:**
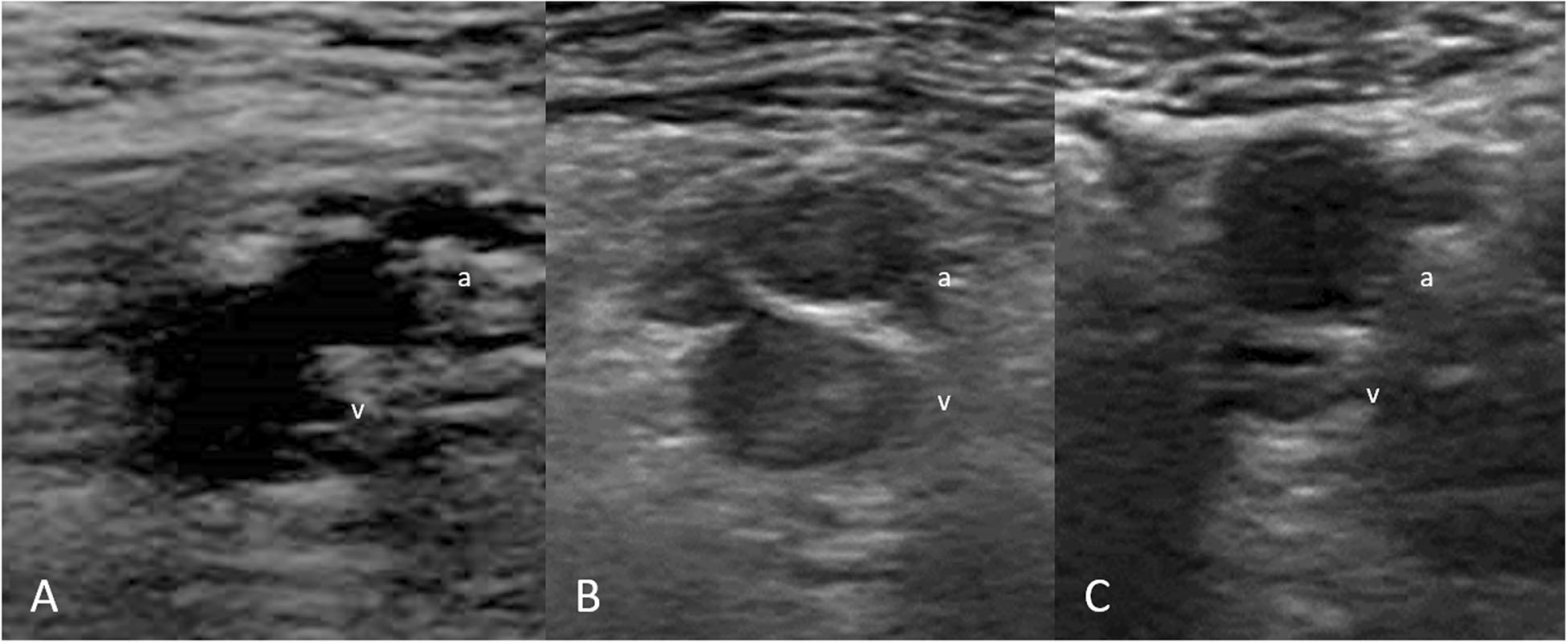
Size of the affected vein: **A** Increased size; The thrombus in the femoral vein (FV) is larger than that in the adjacent superficial femoral artery (SFA). **B** Normal vein size. The thrombus in the FV is equal to the adjacent SFA. **C** Decreased vein size; The thrombus in the FV is smaller than the adjacent SFA. a = superficial femoral artery, v = femoral vein.

**Fig. 2:**
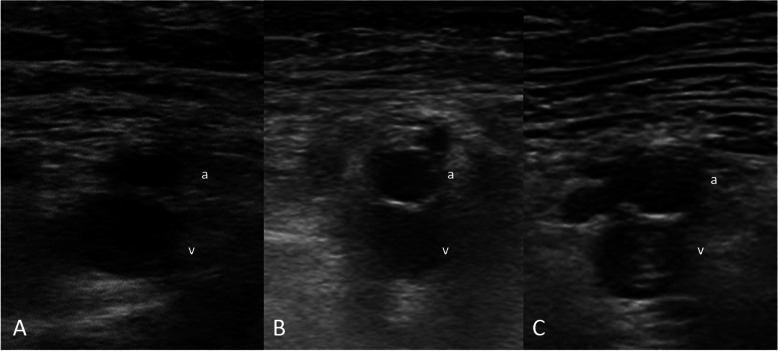
Echogenicity of the thrombus: **A** Anechoic thrombus; The thrombus appears black on the screen. **B** Hypoechoic thrombus; The thrombus appears grey on the screen. **C** Hyperechoic thrombus; The thrombus appears white on the screen. a = superficial femoral artery, v = femoral vein.

**Fig. 3:**
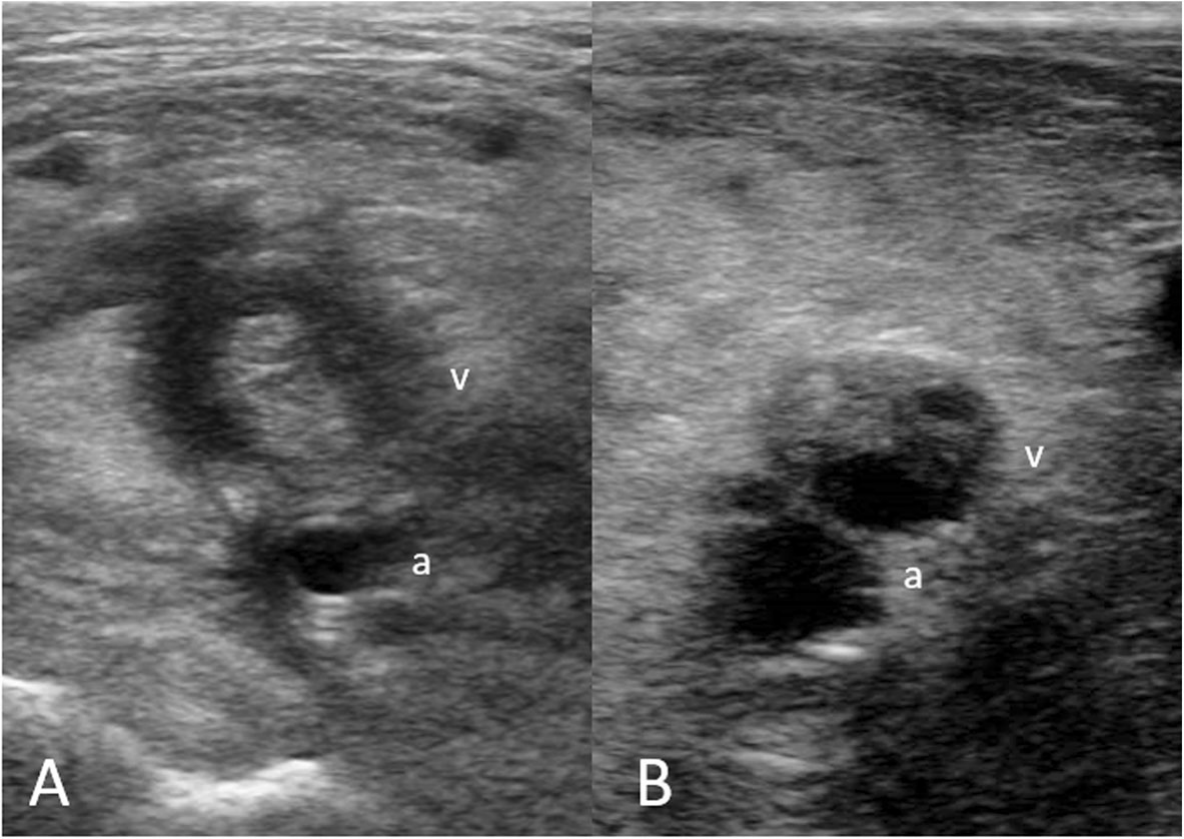
Shape of the thrombus: **A** Concentric thrombus; the thrombus causes total occlusion of the popliteal vein. **B** Eccentric thrombus: the thrombus causes partial occlusion and forms an obtuse angle with the venous wall. a = popliteal artery, v = popliteal vein.

### Patient selection

#### Inclusion criteria

- Lower extremity DVT patients were diagnosed, treated and followed by the venous ultrasound from March 2014 to February 2019.

#### Exclusion criteria

- Patients whose sonographic image were inadequate for interpretation.
- Patients with isolated DVT in the calf vein.
- Patients younger than 18 years old.

### Statistical analysis

Categorical variables and clinical and sonographic characteristics were evaluated. Normally distributed data are presented as the mean and standard deviation. Nonnormally distributed data are presented as the median and range. The comparison was performed by median regression analysis between morphologic findings and the maximum difference in thrombus thickness. Multiple median regression analyses were applied to individual variables (treatment and the timing of the repeat ultrasound). Analytical results are presented as a coefficient and 95% confidence intervals (CI). A *p*-value of < 0.05 was considered statistically significant. STATA version 15.1 (Stata Corp, College Drive, Texas) statistical software was used to analyse the data.

## Results

Between March 2014 and February 2019, 106 lower extremity DVT patients were diagnosed and followed by venous ultrasound. Four patients were excluded from the study due to inadequate sonographic imaging for interpretation, and five patients were excluded due to isolated DVT in the calf vein. Ninety-seven patients were enrolled in the study population.

Out of the 97 patients, 57 (58.8%) patients were male. The age of the patients ranged from 20 to 85 years, with a mean age of 60.4 years. A history of malignancy was the most common risk factor (25.8%), followed by immobility (15.5%), sepsis (2.1%) and trauma (2.1%).

All patients were treated with anticoagulants based on the clinical signs and symptoms before the ultrasound examination. Thirty-seven (38.1%) patients had a history of malignancy, or the clinical signs and symptoms of extensive DVT were treated by low molecular weight heparin (LMWH) 1 mg/kg subcutaneous q 12 h in the initial phase followed by long-term oral vitamin K antagonists (VKAs). Sixty (61.9%) patients were treated with VKAs alone (maintaining INR at 2.0–3.0) (Table 1). The timing of the repeat ultrasound in both groups ranged from 74 to 192 days (the median was 141 days). In the multiple median regression analysis, anticoagulation treatment was significantly associated with the maximum difference in lower extremity DVT thrombus thickness (*P* < 0.001). The adjusted coefficient (95% CI) of LMWH followed by VKAs compared with VKAs alone was − 4 (95% CI: − 4.6, − 3.4). In contrast, the timing of the repeat ultrasound was not significantly associated (*P* value = 0.217) (Table 2).

**Table 1:** Clinical characteristics data of the study population

Characteristics	Sample (***n*** = 97)
**Gender, n (%)**	
Male	57 (58.8)
Female	40 (41.2)
**Age, mean (SD)**	60.4 (46.6)
**Risk factor, n (%)**	
Malignancy	25 (25.8)
Immobility	15 (15.5)
Sepsis	2 (2.1)
Trauma	2 (2.1)
Cardiac arrythmia	1 (1)
Hormonal treatment	1 (1)
Obesity	1 (1)
Pregnancy	0 (0)
**Follow-up time, median (min-max) days**	141 (74–192)
**Treatment, n (%)**	
LMWH followed by VKAs	37 (38.1)
VKAs alone	60 (61.9)

*DVT* Deep vein thrombosis, *LMWH* Low molecular weight heparin, *LMWH* Low molecular weight heparin, *VKAs* Vitamin K antagonists

**Table 2:** Factor associated with maximum difference thickness of the thrombus

Factor	Coef. (95% CI)	***P*** value
**Follow up time**	0.009 (−0.006, 0.025)	0.217
**Treatment**		
Maximum difference thickness in LMWH followed by VKAs	−4 (−4.6, −3.4)	< 0.001*
Maximum difference thickness in VKAs alone	0	

*LMWH* Low molecular weight heparin, *VKAs* Vitamin K antagonists

* *P*-value <0.05 was considered statistically significant

Venous thrombi were predominantly located in the left lower extremities compared with the right lower extremities (62.9 vs 37.1%). The most common location was the popliteal vein (81.4%). However, the maximum thrombus thickness change was found commonly in the proximal femoral vein (32.0%), followed by the common femoral vein (30.9%) and popliteal vein (18.6%). At the first diagnosis, 46.4% of thrombi were found in small veins; 63.9% were hyperechoic thrombi, and 61.9% were eccentric thrombi (Table 3).

**Table 3:** Characteristic data of the venous thrombus

Characteristics	Sample (***n*** = 97)
**Side of cases showing DVT involvement, n (%)**	
Right	36 (37.1)
Left	61 (62.9)
**Anatomic distribution of thrombi, n (%)**	
Common femoral vein (CFV)	62 (63.9)
Proximal femoral vein (prox FV)	72 (74.2)
Mid femoral vein (mid FV)	71 (73.2)
Distal femoral vein (dis FV)	74 (76.3)
Popliteal vein (Pop)	79 (81.4)
**Location at maximum thrombus thickness change, n (%)**	
Common femoral vein (CFV)	30 (30.9)
Proximal femoral vein (prox FV)	31 (32.0)
Mid femoral vein (mid FV)	8 (8.3)
Distal femoral vein (dis FV)	10 (10.3)
Popliteal vein (Pop)	18 (18.6)
**Size of affected vein (relative to adjacent artery), n (%)**	
Increased size	23 (23.7)
Normal size	29 (29.9)
Decreased size	45 (46.4)
**Echogenicity of thrombus**, **n (%)**	
Anechoic	17 (17.5)
Iso/hypoechoic	18 (18.6)
Hyperechoic	62 (63.9)
**Shape of thrombus**, **n (%)**	
Concentric	37 (38.1)
Eccentric	60 (61.9)

*DVT* Deep vein thrombosis

The thrombi found in dilated veins showed a significant decrease in the mean maximum difference in thickness of the thrombus compared with that of thrombi found in small veins: 4 mm vs. 0 mm (Coef. = 3, 95% CI: 1.9, 4.1 and *P*-value < 0.001). Anechoic and hypoechoic thrombi showed a significant decrease in the mean maximum difference in thickness of the thrombus compared with that of hyperechoic thrombi, 5 mm vs 0 mm (Coef. = 4, 95% CI: 3.25, 4.74 and *P*-value < 0.001) and 3 mm vs 0 mm (Coef. = 2, 95% CI: 1.34, 42.66 and *P*-value < 0.001), respectively. Concentric thrombi showed a significant decrease in the mean maximum difference in thickness compared with that of eccentric thrombi: 4 mm vs. 0 mm (Coef. = 2, 95% CI: 1.45, 2.55 and *P*-value < 0.001). Results of median regression analysis between morphologic findings and the maximum difference in thickness of the thrombus after adjustment by the anticoagulant treatment are shown in Table 4.

**Table 4:** Correlation of morphologic sonographic findings and maximum difference in thrombus thickness after adjustment by treatment

Morphologic findings	Maximum difference thickness (min-max) mm	Coef. (95% CI)	***P*** Value
**Size of the affected vein (relative to adjacent artery)**			
Increased size	4 (0–12)	3 (1.90, 4.10)	< 0.001*
Normal size	1 (1–6)	0 (0.00, 0.82)	1.00
Decreased size	0 (0–2)	0	
**Echogenicity of the thrombus**			
Anechoic	5 (0–8)	4 (3.25, 4.74)	< 0.001*
Iso/hypoechoic	3 (0–12)	2 (1.34, 2.66)	< 0.001*
Hyperechoic	0 (0–2)	0	
**Shape of the thrombus**			
Concentric	4 (1–12)	2 (1.45, 2.55)	< 0.001*
Eccentric	0 (0–2)	0	

* P-value <0.05 was considered statistically significant

## Discussion

The results of this study advocate the benefit of venous thrombus morphology classification by ultrasound apart from the first imaging modality for DVT diagnosis. Differences in the size, echogenicity and shape of venous thrombi show different responses to anticoagulant treatment. A concentric anechoic or hypoechoic thrombus, which causes dilatation of the affected vein, shows a statistically significant decrease in thrombus thickness compared with that of eccentric hyperechoic thrombi in retracted veins, which offers minimal to no change in thickness after anticoagulant treatment. No previous clinical studies have reported this issue. Most reviews of venous ultrasound after anticoagulation treatment have focused on the risk of recurrent VTE when residual thrombus occurs [6–8].

With knowledge of the natural history of thrombus formation, ultrasound may specify the age of the thrombus by the difference in morphology. Fibrinogen is converted to fibrin in 1 day, forming a mesh that traps platelets and red blood cells and produces a thrombus. The cross-linking of fibrin and the development of internal zones of haemolysis are responsible for the anechoic-hypoechogenicity of the thrombus. The newly formed thrombus tends to obstruct the vein lumen and expand the venous diameter entirely. The thrombin net squeezes red blood cells and fluid components out of the thrombi during thrombus ageing [9–11]. The proportion of red blood cells in the thrombus decreases with time, and the ultrasound signal intensity is affected by the number of red blood cells in the thrombus. This accounts for the increased echogenic appearance of the aged thrombus [12]. In the following 2–8 weeks, the thrombus organizes or dissolves from balancing clot formation and degradation. A mixture of the hyperplastic response of the endothelium at the site of thrombus deposition and residual thrombus incorporated into the wall during the scarring or fibrotic response causes asymmetrical wall thickening or an eccentric thrombus. The affected vein is often retracted [9–11]. Therefore, an acute obstructing DVT is typically hypoechogenic-concentric shaped, and the vein lumen is dilated. In contrast, the residual thrombus in chronic DVT is often hyperechogenic-eccentric shaped and adheres to one side of the vein wall, and the vein lumen is small.

The thrombus is transformed from being composed predominantly of platelets to having a fibrin composition with replacement by dense connective tissue [13]. Therefore, anticoagulation cannot break apart the chronic thrombus. The results of this study show minimal to no change in thickness (0–2 mm) of eccentric, hyperechoic thrombi in retracted veins. This supports the conclusion that the presence of a chronic thrombus in a symptomatic patient without a new acute thrombus does not require anticoagulant therapy [14].

Although this study shows the different responses to anticoagulants in cases with different venous sizes and echogenicities of thrombi, neither venous size nor thrombi echogenicity reliably distinguishes acute from chronic thrombi [15]. An acute nonobstructing thrombus does not often cause dilatation of the affected vein. Chronic thrombi can be found in dilated veins, especially after the development of venous reflux. The concentric shape of the thrombus remains in the small vein when there is no recanalization. Furthermore, the differentiation of acute and chronic thrombi by morphology remains mainly qualitative and nonstandardized. The ageing of the thrombus and the treatment decision may be purely based on patient reporting of symptoms.

Ultrasound elastography for assessing thrombus stiffness is a quantitative method for determining thrombus age. Few clinical studies involving lower extremity ultrasound have reported the difference between acute and chronic thrombi’s median normalized strain magnitude [16]. The strain ratio of the chronic and subacute thrombosis groups was found to be higher than that of the acute thrombosis group (*P* < 0.001, < 0.05) [17]. Combining thrombus morphology and ultrasound elastography may enhance ultrasound accuracy in thrombus age determination and require further study.

In addition, the limitation of this investigation is that it was a single-centre, retrospective cohort study with a small study population. Approximately 60% of the study population was diagnosed by the clinical manifestation and received LMWH before the ultrasound examination. No recurrent DVT or increase in thrombus thickness was found in our study population. Therefore, the value of differentiating thrombus morphology in follow-up ultrasound after anticoagulant treatment was not discovered.

## Conclusion

The anticoagulant treatment with LMWH followed by VKAs shows a significant decrease in lower extremity DVT thrombus thickness compared with VKAs alone. After adjustment by treatment, the morphologic finding of acute thrombi (concentric, anechoic/hypoechoic and dilated vein) shows a significantly decreased thickness than the morphologic finding of chronic thrombi (eccentric, hyperechoic and retracted vein). Although the chronic thrombi show minimal to no change in thickness, the increase in thickness or recurrent DVT did not occur after anticoagulant treatment.

## Data Availability

Not applicable.

## Abbreviations

VTE: Venous thromboembolism
DVT: Deep venous thrombosis
PE: Pulmonary embolism
PTP: Pretest probability
CFV: Common femoral vein
FV: Femoral vein
Pop V: Popliteal vein
LMWH: Low molecular weight heparin
VKAs: Vitamin K antagonists
